# Novel use of telitacicept in primary immune thrombocytopenia: a case report

**DOI:** 10.1186/s12887-026-06641-9

**Published:** 2026-03-03

**Authors:** Yini Wang, Jia Wang, Bin Hu, Yun Zhou, Yueli Rao, Meiping Lu, Xuefeng Xu

**Affiliations:** 1 Department of Rheumatology Immunology & Allergy, Children’s Hospital, Zhejiang University School of Medicine, National Clinical Research Center for Child Health, Hangzhou, 310052 PR China; 2 Blood transfusion branch of the Joint Service Support Force 903 Hospital, Hangzhou, 310052 PR China

**Keywords:** Telitacicept, primary immune thrombocytopenia, BlyS/APRIL inhibition

## Abstract

**Background:**

Primary immune thrombocytopenia (ITP) is generally a self-limiting disorder in children; however, a subset of cases becomes refractory to conventional treatments or progresses to chronic disease. Managing such refractory instances remains a significant clinical challenge.

**Case Presentation:**

We report the case of a 5-year-old child with refractory ITP that was not adequately controlled with standard therapies, including intravenous immunoglobulin, corticosteroids, and rituximab. Following disease recurrence and limited response to these interventions, the novel biologic agent telitacicept was administered. This intervention led to marked clinical improvement. The diagnostic evaluation, therapeutic course, and successful use of telitacicept are comprehensively detailed.

**Conclusions:**

This case illustrates the potential benefit of telitacicept in the management of pediatric refractory ITP, particularly when conventional treatment options have been exhausted. Further studies are warranted to explore its efficacy and safety in similar challenging cases.

## Background

Primary immune thrombocytopenia (ITP) is an acquired autoimmune hemorrhagic disorder characterized by isolated thrombocytopenia (platelet count < 100 × 10⁹/L) in the absence of other identifiable causes [1]. The annual incidence is estimated at 2–10 cases per 100,000 adults and 5–10 cases per 100,000 children [2, 3]. While pediatric ITP spans the age range of 1 to 18 years, the median age at diagnosis remains concentrated between 5 and 8 years [4, 5]. Based on disease duration, pediatric ITP is categorized as newly diagnosed, persistent, or chronic. Notably, more than 75% of pediatric cases undergo spontaneous remission within one year of diagnosis [6].

The clinical presentation in most children is mild, typically manifesting as bruising, petechiae, or minor epistaxis. These manifestations often follow an acute infection or vaccination [7]. Initial management strategies encompass observation, intravenous immunoglobulin (IVIg), and corticosteroid therapy. For patients with persistent or chronic disease refractory to first-line interventions, second-line therapeutic options include rituximab, thrombopoietin receptor agonists (TPO-RAs), and splenectomy. While conventional treatments effectively manage most patients, a small subset proves exceptionally challenging to treat, demonstrating poor response to standard therapies. These cases fall under the classification of refractory ITP [8]. Telitacicept is a fusion protein that combines TACI with the Fc fragment of human IgG1, targeting BLyS and APRIL to prevent their interaction with all B-cell ligands. Its safety profile in clinical trials is comparable to other B-cell-targeting agents [9]. According to the 2019 American Society of Hematology (ASH) guidelines, refractory immune thrombocytopenia is defined as failure of at least two lines of therapy, including thrombopoietin receptor agonists, with persistent clinically significant thrombocytopenia or bleeding risk [10]. In the present case, the patient failed to achieve sustained remission after corticosteroids, IVIG, rituximab (discontinued due to allergic reaction), and eltrombopag, thus fulfilling criteria for refractory ITP. Following treatment with telitacicept, the patient exhibited significant clinical improvement, demonstrating the potential efficacy of this therapeutic approach in pediatric refractory ITP.

## Case presentation

A 5-year-7-month-old female patient was admitted to the Department of Rheumatology, Immunology and Allergy presenting with a 20-day history of recurrent truncal ecchymoses and epistaxis. Initial laboratory evaluation revealed severe thrombocytopenia with a platelet count of 6 × 10⁹/L (Table 1). Bone marrow aspiration was performed to exclude hematologic malignancy given the severity of thrombocytopenia and atypical clinical presentation. The examination revealed trilineage hyperplasia with impaired platelet production by megakaryocytes, without evidence of malignant infiltration. These findings, alongside the presence of anti-glycoprotein IIb antibodies, confirmed the diagnosis of severe primary immune thrombocytopenia (ITP).

**Table 1:** Laboratory findings at the time of admission. All immunoglobulin measurements were obtained after IVIG

Laboratory indicators	Test value	Reference range
WBC (×10^9^/L)	16.59	4.00–12.00
Hemoglobin (g/L)	134	110–155
Platelet(×10^9^/L)	6	100–400
PCT(%)	0.03	0.07–0.35
CRP (mg/L)	0.43	0.00–6.00
Ferritin(µg/L)	19.2	24.0-336.0
Platelet antibodies(%)	41.7	< 30
ALT (U/L)	27	< 50
AST (U/L)	42	14–44
LDH (U/L)	454	110–295
IgE(IU/ml)	125	0-100
IgG(g/L)	21.95	5-10.6
IgA(g/L)	0.71	0.34–1.38
IgM(g/L)	1.28	0.44–1.34
C3 (g/L)	1.257	0.900–1.800
C4 (g/L)	0.322	0.100–0.400
APTT(seconds)	21.5	23–38
D-dimer(mg/l)	0.13	< 0.55
Fibrinogen(g/L)	2.3	1.69–3.6
CD19(%)	23	10.46–21.77
CD3(%)	68.9	59.5-75.56
CD4(%)	23.55	28.49–41.07
CD8 (%)	31.4	19.7-32.04
NK(%)	8.35	7.83–20.99
ANA	Negative	Negative(<1:100)
anti-ribosomal antibodies	Negative	Negative
anti-dsDNA antibody	Negative	Negative
p-ANCA	Negative	Negative
c-ANCA	Negative	Negative
anti-smith antibody	Negative	Negative
anti-SSA antibody	Negative	Negative
anti-SSB antibody	Negative	Negative

*WBC* White Blood Cell, *PCT* Plateletcrit, *CRP* C-Reactive Protein, *ALT* Alanine Aminotransferase, *AST* Aspartate Aminotransferase, *LDH* Lactate Dehydrogenase, *Ig* Immunoglobulin, *C3* Complement 3, *APTT* Activated partial thromboplastin time, *CD* Cluster of Differentiation, *NK* natural killer, *ANA* antinuclear antibody, anti-*dsDNA* anti-double-stranded DNA, *p-ANCA* perinuclear anti-neutrophil cytoplasmic antibodies, *c-ANCA* cytoplasmic ANCA, *anti-SSA* Anti-Sjögren’s syndrome A

**Fig. 1:**
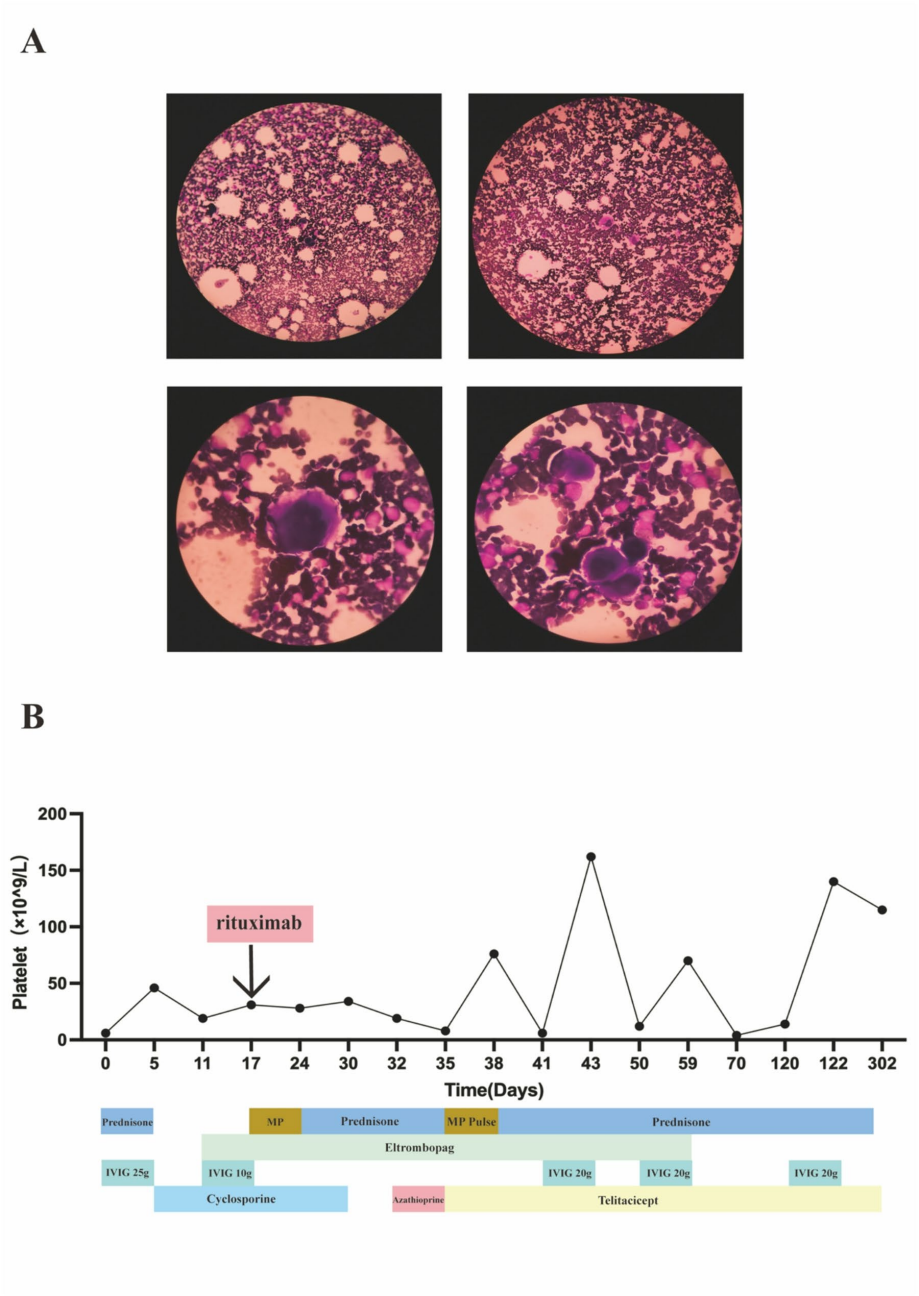
**A** Microscopic image of the patient's bone marrow smear, indicating an increase in megakaryocyte count **B** Overview of treatment course. The graph shows the platelet count since diagnosis, with the corresponding treatment methods shown at the bottom. This indicates that the condition improved after treatment with Telitacicept, with the platelet count gradually increasing

First-line therapy consisted of intravenous immunoglobulin (IVIG, 1 g/kg; patient weight approximately 25 kg) and oral prednisolone acetate (25 mg daily), resulting in partial platelet recovery.Owing to a suboptimal therapeutic response, the regimen was intensified to include cyclosporine (75 mg daily), eltrombopag (25 mg daily), and rituximab (100 mg). However, the rituximab infusion was immediately terminated due to an acute infusion reaction characterized by facial edema, dyspnea, and hypoxemia.The allergic reaction was successfully managed with intramuscular epinephrine and intravenous methylprednisolone, following which maintenance therapy was transitioned to oral prednisone (Fig. 1B).

The patient achieved stable platelet counts by hospital day 24, and was discharged. However, she returned five days later with new-onset headaches. Laboratory findings showed recurrent thrombocytopenia (platelet count: 34 × 10⁹/L) and severe hypertension (BP: 160/110 mmHg), prompting immediate discontinuation of cyclosporine and initiation of azathioprine (25 mg, bid). Despite azathioprine therapy, platelet counts continued to decline. Given the significant adverse effect profile of prolonged azathioprine use, we implemented an aggressive salvage regimen consisting of high-dose methylprednisolone (300 mg/day for 3 days), telitacicept (a BLyS/APRIL inhibitor; 60 mg weekly for 4 doses), Eltrombopag(25 mg, qd) and IVIG (10 g administered on four occasions). This therapeutic strategy successfully induced a robust hematologic response, with platelet counts rising to 70 × 10⁹/L, enabling safe discharge without recurrence of hypertension.

Following discharge, the patient was maintained on a combination therapy of telitacicept (administered weekly) with a carefully tapered prednisone regimen (initial dose: 15 mg/day, gradually reduced to 2.5 mg/day). This protocol initially maintained stable platelet counts. However, coinciding with concurrent viral infections on disease day 70 and 120, the patient developed new-onset petechiae accompanied by thrombocytopenia (platelet count: 14 × 10⁹/L), indicative of disease relapse. Immediate intervention with IVIG resulted in rapid clinical and hematological stabilization, permitting discharge. Subsequently, she continued maintenance telitacicept alongside low-dose prednisone (2.5 mg daily), achieving sustained platelet control. This adjusted regimen successfully maintained durable platelet control, ultimately allowing complete steroid discontinuation (Fig. 1B). The patient currently continues weekly telitacicept therapy with close clinical and laboratory monitoring, demonstrating sustained treatment efficacy.

This study was approved by the Institutional Review Board (IRB) of Children’s Hospital of Zhejiang University, approval number :2025-IRB-0389-P-01.

### Safety monitoring and adverse events

Regarding safety, telitacicept has generally demonstrated an acceptable tolerability profile in clinical trials and real-world studies involving patients with systemic lupus erythematosus and other autoimmune diseases. Reported adverse events mainly include mild to moderate infections (particularly upper respiratory tract infections), transient hypogammaglobulinemia, injection-site reactions, and occasional herpes zoster reactivation [11–13]. Serious infections appear infrequent, although long-term immunologic safety remains under investigation. In the present case, no serious infections, herpes zoster, or progressive hypogammaglobulinemia were observed during telitacicept therapy. Apart from transient viral upper respiratory infections coinciding with disease relapse, no treatment-related adverse events were recorded. Nevertheless, given the limited pediatric experience and short follow-up duration, continued surveillance for infectious and immunologic complications is warranted.

## Discussion

Primary immune thrombocytopenia (ITP) is an immune-mediated disease driven by Fcγ receptor-mediated phagocytosis, characterized by a dual imbalance between premature destruction of platelets in the peripheral blood and inadequate compensatory platelet production in the bone marrow. In this disease, IgG-type autoantibodies in serum target glycoproteins on the platelet surface, primarily including GPIIb-IIIa, GPIb-IX, and GP Ia-IIa. These antibodies activate Fcγ receptors on macrophages in the splenic reticuloendothelial system through the opsonization process, leading to platelet phagocytosis and destruction. Additionally, homologous antibodies cross-react with corresponding glycoproteins on bone marrow megakaryocytes, triggering antibody-dependent cell-mediated cytotoxicity (ADCC) and interfering with megakaryocyte maturation and platelet release.ITP exhibits significant clinical heterogeneity, ranging from self-limiting to chronic, with underlying mechanisms extending beyond the classical antibody pathway. Specifically, molecular mimicry (pathogen antigens mimicking platelet epitopes), T/B cell clonal abnormalities (such as TCR/BCR repertoire skewing and apoptosis resistance), and oxidative stress (reactive oxygen species damaging platelet membrane phospholipid symmetry and exposing phagocytic signal phosphatidylserine) collectively constitute the multidimensional pathological network of ITP [14–16].

BLyS (B-lymphocyte stimulating factor) and APRIL (apoptosis-related protein 4 ligand) are key factors regulating B cell survival, differentiation, and maturation [17, 18]. In patients with immune thrombocytopenia (ITP), the overexpression of these two factors leads to abnormal clonal expansion of autoreactive B cells and enhanced differentiation of surviving plasma cells, thereby further triggering the sustained production of pathological anti-platelet antibodies (IgG). Telitacicept, as a recombinant fusion protein (TACI-Fc), dual-neutralizes BLyS and APRIL, thereby inhibiting the abnormal activation of autoreactive B cells and antibody production at the source, while also regulating T cell function and the immune microenvironment. Emerging evidence supports the efficacy of telitacicept in refractory autoimmune cytopenias, particularly in systemic lupus erythematosus–associated thrombocytopenia and nephritis [19–21]. Recent studies have demonstrated favorable safety and clinical responses in both adult and pediatric lupus populations [22–24], suggesting broader immunomodulatory potential through dual BLyS/APRIL blockade. This mechanism simultaneously improves peripheral platelet destruction (mediated by antibody/FcγR-mediated phagocytosis) and bone marrow suppression (megakaryocyte damage) in ITP patients. Its mechanism of action overcomes the limitations of traditional therapies, offering a new immunomodulatory strategy for refractory or chronic ITP. It should be acknowledged that telitacicept was administered concomitantly with high-dose corticosteroids and repeated IVIG, confounding the ability to establish a definitive causal link between telitacicept administration and the observed platelet recovery. Therefore, the observed hematologic response represents a temporal association in a complex salvage regimen rather than proof of isolated drug efficacy. However, data regarding telitacicept use in pediatric ITP remain extremely limited, and long-term safety in children has not been established. Larger prospective studies are required before routine pediatric application can be recommended.

## Conclusion

This case describes a temporally associated platelet response following telitacicept-containing salvage therapy in a child with refractory immune thrombocytopenia. Given the concomitant use of corticosteroids and IVIG, these findings should be considered hypothesis-generating rather than confirmatory. While telitacicept may represent a potential therapeutic option in highly refractory pediatric ITP, its efficacy and safety require validation in controlled prospective studies. 

## Data Availability

The data will be available on reasonable request to the corresponding author.

## Abbreviations

ITP: Primary Immune Thrombocytopenia
IVIg: Intravenous Immunoglobulin
TPO-RAs: Thrombopoietin Receptor Agonists
BLyS: B-lymphocyte Stimulator
APRIL: A Proliferation-Inducing Ligand
TACI: Transmembrane Activator and CAML Interactor
IgG: Immunoglobulin G
ADCC: Antibody-Dependent Cell-Mediated Cytotoxicity
SLE: Systemic Lupus Erythematosus
RA: Rheumatoid Arthritis
MG: Myasthenia Gravis
qd: quaque die
bid: bis in die
BP: Blood Pressure
